# Early detection of immune checkpoint inhibitor-related subclinical cardiotoxicity: A pilot study by using speckle tracking imaging and three-dimensional echocardiography

**DOI:** 10.3389/fcvm.2022.1087287

**Published:** 2022-12-21

**Authors:** Aiqing Xu, Ming Yuan, Xiaoping Zhan, Gangjian Zhao, Guanyu Mu, Tingting Wang, Hailong Hu, Huaying Fu

**Affiliations:** ^1^Tianjin Key Laboratory of Ionic-Molecular Function of Cardiovascular Disease, Department of Cardiology, Tianjin Institute of Cardiology, Second Hospital of Tianjin Medical University, Tianjin, China; ^2^Department of Cardiology, Tianjin Medical University General Hospital, Tianjin, China; ^3^Department of Urology, Second Hospital of Tianjin Medical University, Tianjin, China

**Keywords:** subclinical cardiotoxicity, echocardiography, strain, TAPSE, ICIs

## Abstract

**Background:**

Early detection of subclinical cardiotoxicity of immune checkpoint inhibitor (ICI) therapy can be challenging.

**Objective:**

To evaluate subclinical cardiac dysfunction using two-dimensional speckle tracking imaging (2D-STI) and three-dimensional echocardiography in Chinese patients.

**Methods:**

Fifty-five consecutive patients with malignant tumors treated by immunotherapy were included. They were examined by echocardiography before immunotherapy and after immunotherapy. Left ventricular ejection fraction (LVEF) was calculated in three-dimensional imaging. Moreover, left ventricular global longitudinal peak systolic strain (LVGLS), left ventricular global circumferential peak systolic strain (LVGCS), right ventricular global longitudinal systolic strain (RVGLS), right ventricular free wall longitudinal peak systolic strain (RVFWLS), and tricuspid annular plane systolic excursion (TAPSE) were evaluated. Clinical and laboratory parameters were recorded. Cardiac toxicity events were defined as the presence of heart failure symptoms, LVEF reduction, and increase in troponin. Subclinical cardiac toxicity was defined as cardiac dysfunction associated with ICI treatment, with absent or delayed ICI-associated cardiotoxicity clinical symptoms.

**Results:**

Compared with baseline, the LVGLS, TAPSE, and RVGLS significantly deteriorated after ICI treatment [(–18.63 ± 2.53)% vs. (–17.35 ± 2.58)%, *P* = 0.000; 18.29 ± 6.23 vs. 14.57 ± 3.81, *P* = 0.0001; and (–18.45 ± 4.65)% vs. (–14.98 ± 3.85)%, *P* = 0.0001, respectively]. LVGLS (–17.35 ± 2.58, *P* = 0.000), TAPSE (14.57 ± 3.81, *P* = 0.0001), and RVGLS [(–14.98 ± 3.85)%, *P* = 0.0001] were decreased after ICI immunotherapy. Kaplan-Meier curve analysis showed that LVGLS was more sensitive than the cardiac toxicity events to assess ICI-related subclinical cardiac dysfunction (log-rank *P* = 0.205). The ROC curve showed that the cutoff value of ΔLVGLS was -13%.

**Conclusion:**

Subclinical cardiac dysfunction can be detected using two-dimensional speckle-tracking imaging. LVGLS, RVGLS, and TAPSE are more sensitive indices for detection.

**Clinical trial registration:**

[https://www.chictr.org.cn/showprojen.aspx?proj=27498], identifier [ChiCTR1800016216].

## 1 Introduction

Immune checkpoint inhibitors (ICIs) including PD1/PDL1 were the novel immunotherapy drugs for various cancers; however, early detection of subclinical cardiotoxicity ICI therapy has been challenging (1). With few recent exceptions, the vast majority of articles on the toxicities of checkpoint inhibitors have underestimated or even neglected cardiac toxicity (2). According to reports, PD1/PDL1 immunotherapy can lead to myocardial damage, and cardiotoxicity can be often fatal and is associated with a poor prognosis (3). Thus, continuous monitoring during immunotherapy for early detection of left ventricular (LV) dysfunction is necessary.

Echocardiography enables the identification of left ventricular dysfunction, cardiac valves, and pericardial diseases and is one of the most important clinical tools in the diagnosis and management of myocardial damage. However, the traditional ultrasound indices are not sufficiently sensitive for the detection of early changes in cardiac function (4). Two-dimensional strain parameters, especially left ventricular longitudinal strain, have been shown to precede a decrease in left ventricular ejection fraction (LVEF) during PD1/PDL1 immunotherapy (5). Global longitudinal strain (GLS) has been recommended as a sensitive index by ASE/EACI and ESC guidelines during the follow-up of chemotherapy (6, 7). In this study, we evaluated the cardiac dysfunction associated with ICI treatment in Chinese patients by using speckle-tracking imaging and three-dimensional echocardiography.

## 2 Materials and methods

### 2.1 Study population

The study protocol was approved by the medical Ethical Review Boards of the Tianjin Medical University Second Hospital (number: KY2018K090). The study was registered with the Chinese Clinical Trial Registry (registration number: ChiCTR18000162216). All participants provided informed consent. A total of 55 consecutive patients hospitalized in the Urology department and Oncology Department of Tianjin Medical University Second Hospital from June 2018 to October 2020 who were naive to ICI treatment were enrolled in the study.

The inclusion criteria included the following: (1) 18–90 years old, (2) cancer confirmed by histopathological results, and (3) normal liver and renal function.

The exclusion criteria were patients with existing (1) heart failure, (2) myocardial infarction, (3) myocarditis, (4) cardiomyopathy, (5) severe valvular diseases, (6) patients who were pregnant or were lactating, (7) patients with poor echocardiography image qualities, and (8) those with less than 6 months survival.

### 2.2 Laboratory characteristics and clinical history

Laboratory findings included cardiac enzymes [troponin, creatine kinase (CK), and creatine kinase MB (CKMB)], blood routine test (hemoglobin and platelet levels), liver function [albumin, alanine transaminase (ALT), aspartate aminotransferase (AST), and total bilirubin], renal function (urea, creatinine, and uric acid), glucose and thyroid function [triiodothyronine (T3), tetraiodothyronine (T4), and thyroid-stimulating hormone (TSH)], and clinical history including gender, age, height, weight, blood pressure, heart rate, hypertension, diabetes mellitus, heart diseases, smoking, and alcohol intake history, which were recorded at baseline. Transthoracic echocardiograms were performed before immunotherapy, and pre-ICI and after ICI administration.

### 2.3 Transthoracic echocardiography

The echocardiographic images were performed in the left recumbent position of the patients. Echocardiographic measurements were based on current guidelines for assessing cardiovascular structure and function. All patients underwent transthoracic echocardiography using a Philips IE33 ultrasound system equipped with an X5-1 (2.5–3.5 MHz) probe. Images of at least five cycles of sinus rhythm were digitally stored in the original Digital Imaging and Communications in Medicine format for offline analysis. The images were recorded in the following standard views: parasternal long-axis view, parasternal short-axis view, apical four-chamber view, apical three-chamber view, and apical two-chamber view.

### 2.4 General two-dimensional echocardiography parameters

Left atrial diameter (LAD), left ventricular end-diastolic diameter (LVEDD), left ventricular end-systolic diameter (LVESD), left ventricle posterior wall thickness (LVPWT), and interventricular septum thickness (IVST) were measured in the parasternal long-axis view. The right ventricular area change rate (RVFAC) was calculated *via* the maximum and minimum areas of the right ventricle [(RVAmax–RVAmin)/RVAmax]. The tricuspid annular plane systolic excursion (TAPSE) was measured in the M mode of the four-chamber view. Mitral inflow velocities and mitral annulus velocities were obtained by pulsed and tissue Doppler echocardiography, respectively. Left atrial volume (LAV) was measured in three-dimensional echocardiography.

### 2.5 Two-dimensional strain parameters and three-dimensional echocardiography

The QLAB 10.8 software was used to analyze the images offline. The left ventricle was divided into 17 myocardial segments, and the GLS was calculated as the average longitudinal strain at the end of the systole. The left ventricular global circumference strain (GCS) was measured in the parasternal short-axis views (SAX B, SAX M, and SAX A) according to the software instructions. The right ventricular longitudinal strain and free-wall longitudinal strain were measured accordingly at AP4 view. Left ventricular end-systolic volume (LVESV), left ventricular end-diastolic volume (LVEDV), and 3D LVEF were calculated in three-dimensional echocardiography imaging of a four-chamber view.

### 2.6 Definition of cardiac toxicity and subclinical cardiac toxicity

Cardiac toxicity was defined as LVEF less than 53% or decreasing more than 10% in LVEF or 15% in GLS when compared with the baseline value (6, 8). In addition, cardiac toxicity was also defined as a >5% decrease in LVEF along with the presence of heart failure symptoms, troponin increased extending normal range combined with the patient having the sign of myocarditis, cardiovascular death, cardiogenic shock, and cardiac arrest. TAPSE less than 16 mm represented right ventricular systolic dysfunction (9). Subclinical cardiac toxicity was defined as early changes in left ventricular and right ventricular mechanics after ICI treatment, but clinical symptoms of ICI-associated were absent or happened later. The cutoff follow-up time was 220 days. According to reports, the cutoff of LVGLS, RVGLS, and RVFWLS was –18, –17.3, and –19.5%, respectively (10–12).

### 2.7 Statistical analysis

The IBM SPSS statistics 20.0 software (IBM Corporation, Armonk, NY, USA) was used for statistical analysis. All continuous variables were expressed as means ± standard deviation and compared by paired *t*-test. Categorical data were presented as numbers (%) and compared through the chi-square test. The data were analyzed using two-tailed tests, and *P* < 0.05 indicated that the difference was statistically significant. Subgroup analysis was performed. Cox regression was employed for survival analysis. ICI-related cardiotoxicity occurred time and survival cumulative percentage was analyzed through the Kaplan–Meier curve, and the *P*-value was calculated by log-rank test. The receiver operator characteristic (ROC) curve was done for analyzing LVGLS.

## 3 Results

### 3.1 Patient clinical characteristics

In total, 55 patients met the criteria of this study. Among them, 40 (72.3%) were male patients with a mean age of 62, 17 patients had hypertension, nine patients had diabetes mellitus, five patients had a history of cardiovascular diseases, and 23 patients had a smoking history. Their cardiac enzymes (troponin, CK, and CKMB), routine blood results (hemoglobin and platelet), and liver and renal function (albumin, ALT, AST, total bilirubin, urea, creatinine, and uric acid) were within normal ranges. The baseline clinical data of the patients are shown in Table 1. All patients had a normal left ventricular ejection fraction of more than 53% at the baseline. The most common indications for ICIs were urinary system malignancies and lung cancer (87.3%) (Table 1). Patients were more likely to have received single ICI therapy and later combination ICI therapy with chemotherapy, and clinical laboratory results are shown in Table 2. Compared with the pre-ICI data, all the aforementioned clinical laboratory results were similar to the post-ICI data, except for the AST parameter, though AST was in the normal range either before or after ICI treatment.

**TABLE 1:** Baseline characteristics of the overall group.

Overall group (*n* = 55)			
Baseline characteristics		Cancer sites	
Male, %	40 (72.3)	Bladder, %	33 (60.0)
Age, years	62 ± 12	Kidney, %	5 (9.1)
Height, m	1.68 ± 0.07	Renal pelvis, %	4 (7.3)
Weight, kg	65.07 ± 9.33	Ureters, %	3 (5.5)
BMI, kg/m^2^	22.94 ± 2.77	Lung, %	3 (5.5)
BSA, m^2^	1.70 ± 0.15	Others, %	7 (12.7)
sBP, mmHg	128 ± 11	**Combination treatment**	
dBP, mmHg	78 ± 8	Taxol, %	19 (34.5)
HR, bpm	79 ± 8	Gemcitabine, %	13 (23.6)
HTN, %	17 (31)	Cisplatin, %	12 (21.8)
DM, %	9 (16.4)	Pemetrexed, %	2 (3.6)
Heart disease, %	5 (9)	Carboplatin, %	2 (3.6)
Smoke, %	23 (42)	Nedaplatin, %	1 (1.8)
Alcohol intake, %	9 (16)	Oxaliplatin, %	2 (3.6)
**Medication**		Tegafur, %	1 (1.8)
ACEI/ARB, %	5 (9)	Fluorouracil, %	1 (1.8)
β-blocker, %	2 (3.6)	Docetaxel, %	2 (3.6)
CCB, %	9 (16.3)	Etoposide, %	1 (1.8)
**Cancer type**		Dacarbazine, %	1 (1.8)
Urothelial carcinoma, %	34 (61.8)	Targeted drugs, %	8 (14.5)
Adenocarcinoma, %	2 (3.6)	**ICIs therapy**	
Squamous carcinoma, %	3 (5.4)	PD-1	49 (89.1)
Clear cell carcinoma, %	4 (7.3)	PD-L1	6 (10.9)
Else, %	12 (21.8)	CTLA-4	0 (0)

Values are mean ± standard deviation or %(*n*); BMI, body mass index; BSA, body surface area; sBP, systolic blood pressure; dBP, diastolic blood pressure; HR, heart rate; bpm, beats per minute; HTN, hypertension; DM, diabetes mellitus; ACEI, angiotensin-converting enzyme inhibitors; ARB, angiotensin receptor blocker; CCB, calcium channel blockers. Equation: BMI = weight/(height*height); LAVI = LAV/BSA; BSA = 0.61*height + 0.0128*weight -0.1529.

**TABLE 2:** Patients’ clinical laboratory data before and after immune checkpoint inhibitors (ICIs) treatment.

Clinical laboratory parameters	Pre-ICIs	Post-ICIs	*P*-value
Troponin, ng/ml	0.02 ± 0.02	0.03 ± 0.14	0.356
CK, U/L	62.30 ± 45.45	70.11 ± 55.69	0.094
CKMB, U/L	13.82 ± 6.09	14.03 ± 6.79	0.810
HGB, g/L	119.38 ± 27.27	120.24 ± 26.26	0.837
PLT, 10^9^/L	249.66 ± 72.61	247.64 ± 78.49	0.831
K, mmol/L	4.37 ± 0.38	4.30 ± 0.45	0.278
Na, mmol/L	141.47 ± 4.14	140.96 ± 4.52	0.387
Cl, mmol/L	105.78 ± 4.07	103.59 ± 15.02	0.287
ALB, g/L	41.69 ± 4.67	42.62 ± 4.89	0.196
ALT, U/L	20.30 ± 14.18	24.69 ± 19.38	0.130
AST, U/L	19.00 ± 6.95	23.01 ± 12.62	0.018*
TBIL, μmol/L	8.63 ± 2.69	9.66 ± 5.64	0.152
GLU, mmol/L	5.78 ± 1.82	5.47 ± 1.89	0.253
Urea, mmol/L	6.11 ± 2.08	6.23 ± 1.89	0.588
Cr, μmol/L	89.75 ± 37.64	86.58 ± 31.23	0.388
UA, μmol/L	359.12 ± 116.02	354.62 ± 101.64	0.726
T3, ng/ml	1.64 ± 0.52	1.60 ± 0.61	0.895
T4, μg/dl	91.08 ± 34.98	99.09 ± 19.41	0.185
TSH, μIU/ml	1.70 ± 5.30	3.99 ± 9.24	0.092

CK, creatine kinase; CKMB, creatine kinase isoenzyme; HGB, hemoglobin; PLT, blood platelet; ALB, albumin; ALT, alanine transaminase; AST, aspartate transaminase; TBIL, total bilirubin; Glu, glucose; Cr, creatinine; UA, uric acid; TSH, thyroid-stimulating hormone; *P < 0.05.

### 3.2 Echocardiography results

**FIGURE 1:**
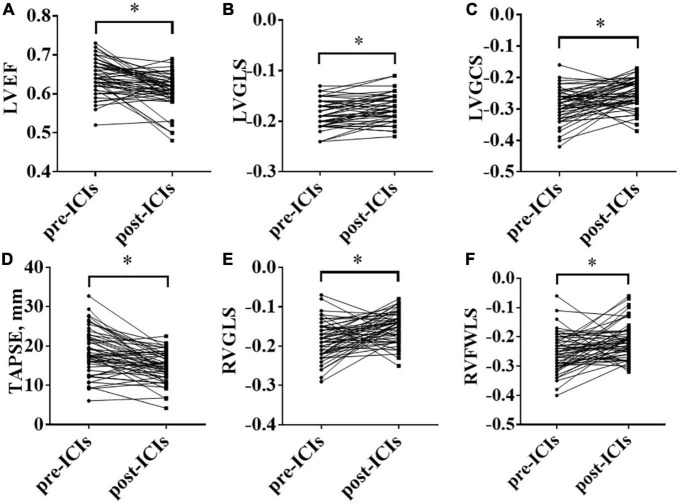
Changes in left ventricular ejection fraction (LVEF), left ventricular global longitudinal peak systolic strain (LVGLS), left ventricular global circumferential peak systolic strain (LVGCS), tricuspid annular plane systolic excursion (TAPSE), right ventricular global longitudinal systolic strain (RVGLS), and right ventricular free-wall longitudinal peak systolic strain (RVFWLS) before and after immune checkpoint inhibitors (ICIs) treatment; **(A)**: LVEF; **(B)**: LVGLS; **(C)**: LVGCS; **(D)**: TAPSE; **(E)**: RVGLS; **(F)**: RVFWLS; *P < 0.05.

Compared with the pre-ICI data, RVFAC [(52.16 ± 12.18)% vs. (48.62 ± 9.84)%, *P* = 0.064] had no significant change after ICI treatment. mPAP (16.77 ± 8.71 vs. 22.03 ± 9.08, *P* = 0.0001), IVST (9.15 ± 1.86 vs. 9.74 ± 1.95, *P* = 0.004), LVEDD (46.03 ± 4.06 vs. 47.27 ± 3.59, *P* = 0.01), LVEDS (25.47 ± 3.86 vs. 27.39 ± 4.34, *P* = 0.002), LVPWT (8.75 ± 1.41 vs. 9.39 ± 1.41, *P* = 0.0001), E/e’ ratio (10.09 ± 2.51 vs. 11.04 ± 2.45, *P* = 0.011), LAD (35.09 ± 4.93 vs. 35.87 ± 4.63, *P* = 0.038), and LAV (30.40 ± 10.17 vs. 34.79 ± 12.32, *P* = 0.005) were significantly increased and LV3DEF [(60.89 ± 11.77)% vs. (57.00 ± 8.18)%, *P* = 0.022] was significantly decreased, while they were in normal range either before or after ICI treatment. Compared with the baseline data, the LVEF, LVGLS, LVGCS, TAPSE, RVGLS, and RVFWLS were significantly worsened after ICI treatment [(64.49 ± 4.36)% vs. (60.75 ± 4.43)%, *P* = 0.0001; (–18.63 ± 2.53)% vs. (–17.35 ± 2.58)%, *P* = 0.000; (–28.83 ± 5.09)% vs. (–24.98 ± 4.51)%, *P* = 0.0001; 18.29 ± 6.23 vs. 14.57 ± 3.81, *P* = 0.0001; (–18.45 ± 4.65)% vs. (–14.98 ± 3.85)%, *P* = 0.0001; and (–25.49 ± 6.42)% vs. (–21.33 ± 6.00)%, *P* = 0.001, respectively] (Figure 1). LVEF, LVGCS, and RVFWLS were in the normal range either before or after ICI treatment. LVGLS (–17.57 ± 2.65, *P* = 0.001), TAPSE (14.57 ± 3.81, *P* = 0.0001), and RVGLS (–14.98 ± 3.85%, *P* = 0.0001) were below their normal range individually after ICI immunotherapy. Echocardiography results are shown in Table 3. Subgroup analysis of LVGLS showed similar results in age, gender, and drug subgroups (Table 4).

**TABLE 3:** Patients’ echocardiography data before and after immune checkpoint inhibitors (ICIs) treatment.

	Pre-ICIs	Post-ICIs	t value	*P*-value
**Echocardiography parameter**				
RVFAC, %	52.16 ± 12.18	48.62 ± 9.84	1.892	0.064
mPAP, mmHg	16.77 ± 8.71	22.03 ± 9.08	-3.904	0.0001*
TAPSE, mm	18.29 ± 6.23	14.57 ± 3.81	4.709	0.0001*
IVST, mm	9.15 ± 1.86	9.74 ± 1.95	-3.009	0.004*
LVEDD, mm	46.03 ± 4.06	47.27 ± 3.59	-2.655	0.01*
LVEDS, mm	25.47 ± 3.86	27.39 ± 4.34	-3.324	0.002*
LVPWT, mm	8.75 ± 1.41	9.39 ± 1.41	-3.705	0.0001*
E/e’ ratio	10.09 ± 2.51	11.04 ± 2.45	-2.647	0.011*
LVEF, %	64.49 ± 4.36	60.75 ± 4.43	5.174	0.0001*
LV3DEF, %	60.89 ± 11.77	57.00 ± 8.18	2.352	0.022*
LAD, mm	35.09 ± 4.93	35.87 ± 4.63	-2.127	0.038*
LAV, ml	30.40 ± 10.17	34.79 ± 12.32	-2.903	0.005*
**Right ventricular strain**				
RVGLS, %	-18.45 ± 4.65	-14.98 ± 3.85	-4.184	0.0001*
RVFWLS, %	-25.49 ± 6.42	-21.33 ± 6.00	-3.689	0.001*
**Left ventricular strain**				
LVGLS, %	-18.63 ± 2.53	-17.35 ± 2.58	-4.848	0.000*
LVGCS, %	-28.83 ± 5.09	-24.98 ± 4.51	-4.692	0.0001*

RVFAC, right ventricular fractional area change; mPAP, mean pulmonary artery blood pressure; TAPSE, Tricuspid annular plane systolic excursion; IVST, interventricular septum thickness; LVEDD, left ventricular end-diastolic dimension; LVESD, left ventricular end-systolic dimension; LVPWT, left ventricular posterior wall thickness; E/e’ ratio, the ratio of early diastolic left ventricular filling peak velocity (E) and early peak mitral annular velocity (e’); LVEF, left ventricular ejection fraction; LV3DEF, left ventricular three-dimensional ejection fraction; LAD, left atrial diameter; LAV, left atrial volume; RVGLS, right ventricular global longitudinal peak systolic strain; RVFWLS, right ventricular free wall longitudinal peak systolic strain; LVGLS, left ventricular global longitudinal peak systolic strain; LVGCS, left ventricular global circumferential peak systolic strain; *P < 0.05.

**TABLE 4:** Subgroup analysis of echocardiography data before and after immune checkpoint inhibitors (ICIs) treatment.

	Subgroup	Pre-ICIs	Post-ICIs	t value	*P*-value
TAPSE, mm	Female (*n* = 15)	17.63	13.87	2.708	0.017*
	Male (*n* = 40)	19.01	14.85	4.791	0.000*
LVGLS, %	Female (*n* = 15)	-18.53	-17.33	-1.903	0.078
	Male (*n* = 40)	-18.67	-17.36	-2.897	0.000*
RVGLS, %	Female (*n* = 15)	-19.13	-15.67	-1.804	0.093
	Male (*n* = 40)	-18.20	-14.73	-3.851	0.000*
TAPSE, mm	<65 years (*n* = 33)	18.56	14.66	3.824	0.001*
	≥65 years (*n* = 22)	18.73	14.45	4.136	0.000*
LVGLS, %	<65 years (*n* = 33)	-18.67	-17.48	-2.569	0.002*
	≥65 years (*n* = 22)	-18.57	-17.14	-2.313	0.002*
RVGLS, %	<65 years (*n* = 33)	-18.52	-14.55	-4.502	0.000*
	≥65 years (*n* = 22)	-18.36	-15.64	-1.689	0.106
TAPSE, mm	Single (*n* = 24)	18.33	14.50	3.651	0.001*
	Combined (*n* = 31)	18.85	14.63	4.133	0.000*
LVGLS, %	Single (*n* = 24)	-17.88	-17.08	-0.634	0.034*
	Combined (*n* = 31)	-19.23	-17.57	-4.475	0.000*
RVGLS, %	Single (*n* = 24)	-17.96	-15.42	-1.740	0.095
	Combined (*n* = 31)	-18.84	-14.65	-4.437	0.000*

TAPSE, tricuspid annular plane systolic excursion; LVGLS, left ventricular global longitudinal peak systolic strain; RVGLS, right ventricular global longitudinal peak systolic strain. *P < 0.05.

### 3.3 Prognosis analysis by Kaplan–Meier curve

**FIGURE 2:**
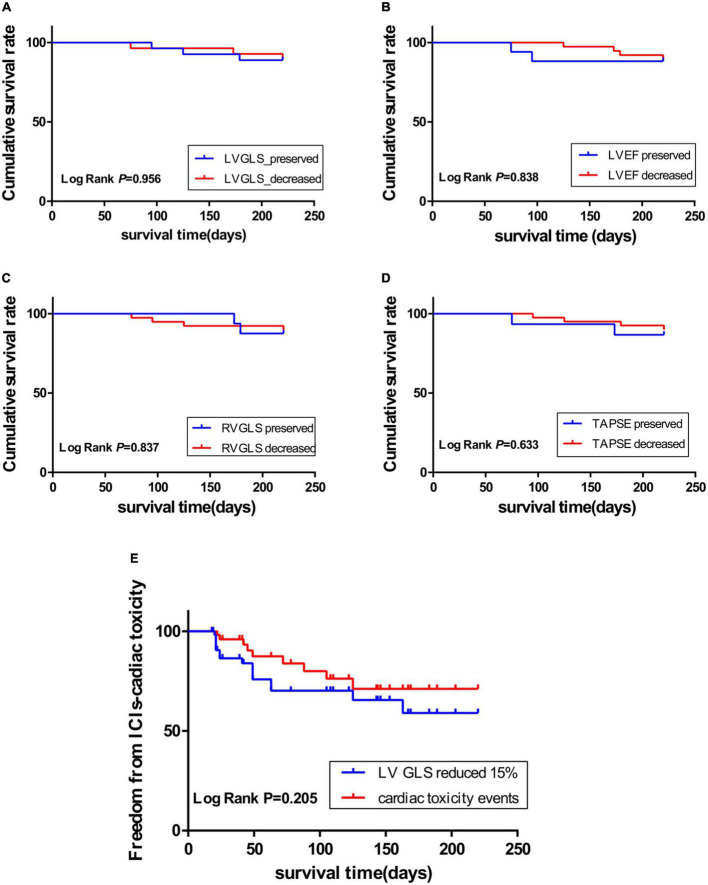
Kaplan–Meier curve showing the cumulative survival rate according to decreased or preserved left ventricular global longitudinal peak systolic strain (LVGLS), left ventricular ejection fraction (LVEF), right ventricular global longitudinal systolic strain (RVGLS), and tricuspid annular plane systolic excursion (TAPSE); **(A)**: LVGLS; **(B)**: LVEF; **(C)**: RVGLS; **(D)**: TAPSE. **(E)**: Kaplan–Meier curve showing the immune checkpoint inhibitor (ICI)-related cardiotoxicity survival time through LVGLS reduced by more than 15% (blue curve), and events (red curve) including heart failure symptoms, troponin increased exceeding the normal range, and LVEF reduced by more than 10% and less than 53%.

**FIGURE 3:**
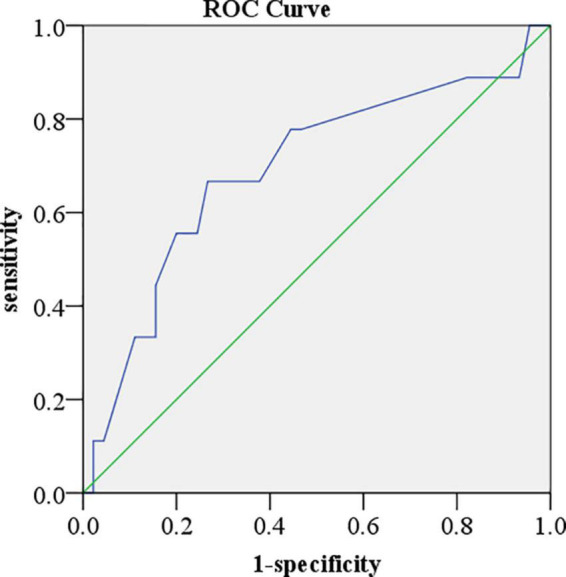
Receiver operator characteristic curve for analyzing left ventricular global longitudinal peak systolic strain (LVGLS). The area under the receiver operator characteristic (ROC) curve was 0.693. The cutoff value of ΔLVGLS was –13%.

Fifty-five patients enrolled in the present study were followed up for 220 days. Six (10.9%) patients died, 15 (27.3%) patients had more than 15% LVGLS decrease, 10 (18.2%) patients had cardiac toxicity (LVEF reduced by 10% and less than 53%), and four (7.3%) patients developed interstitial pneumonia. The KM curve analysis showed that the standard of LVGLS decreased by more than 15% and was more sensitive than the cardiac toxicity events to assess ICI-related subclinical cardiac toxicity, but there was no statistical significance (log-rank *P* = 0.205) (Figure 2). Kaplan–Meier curve analysis demonstrated that patients with decreased LVEF, LVGLS, RVGLS, and TAPSE tended to have higher rates of long-term mortality, although statistical significance was not reached (Figure 2). Cox regression analysis clarified two factors significantly related to the survival rates, including the single or combined treatment and LVGLS decreased or preserved (Table 5). The ROC curve for analyzing the LVGLS showed that the area under the ROC curve was 0.693, and the cutoff value of ΔLVGLS was –13% (Figure 3).

**TABLE 5:** Cox regression analysis for survival analysis.

Factors	*P*-value
Age	0.459
Gender	0.126
Single or combined treatment	0.039*
TAPSE decreased/preserved	0.434
RVGLS decreased/preserved	0.484
LVGLS decreased/preserved	0.045*

TAPSE, tricuspid annular plane systolic excursion; LVGLS, left ventricular global longitudinal peak systolic strain; RVGLS, right ventricular global longitudinal peak systolic strain. *P < 0.05.

## 4 Discussion

The present study showed that LVGLS, RVGLS, and TAPSE were significantly reduced to less than the normal range after ICI administration. Decreased LVGLS reflected left ventricular dysfunction while RVGLS and TAPSE reflected right ventricular dysfunction. By contrast, other parameters including LVEF, LVEDD, LVEDS, and RVFAC were within normal limits following ICI immunotherapy. Thus, it would appear that LVGLS, RVGLS, and TAPSE are more sensitive indices for the early detection of ICI-related subclinical myocardial toxicity.

### 4.1 ICI-associated cardiac dysfunction

Recent reports have expanded the effect of ICI therapy on the cardiovascular system to include an increase in cardiac dysfunction without myocarditis, arrhythmias, venous thromboembolic disease, accelerated atherosclerosis, and atherosclerosis-related cardiovascular events (13). The risk factors associated with ICI-related cardiotoxicity include male sex, sleep apnea, higher BMI, history of radiation, and low neutrophil-to-lymphocyte ratios (14, 15). However, single ICI did not increase the risk of cardiotoxicity compared with chemotherapy and combination immunotherapy did not increase the risk of cardiotoxicity compared with single ICI (16). The association between immune checkpoint inhibitor therapy and an increase in cardiovascular events is not only limited to events occurring within the first few weeks after starting therapy but also can include events that occur months to years after therapy (13). The median time from treatment initiation to clinical manifestation of ICI-related events was 30 days for myocarditis, 30 days for pericardial disorders, and 55 days for vasculitis (17). The current gold standard for myocarditis diagnosis is histological findings of endomyocardial lymphocyte infiltration with myocyte necrosis (18). Transthoracic echocardiogram (TTE) and elevated cardiac biomarkers, including serum troponin, CK-MB, total CK, and natriuretic peptide levels are important tests in the evaluation of suspected myocarditis (19). TTE can also detect other cardiac manifestations, including pericardial effusion or pericardial thickening and intra-cardiac thrombi (19). However, there were no standard examinations to detect ICI-associated cardiac dysfunction. Our study found that LVGLS, RVGLS, and TAPSE were more sensitive indices for the early detection of ICI-related subclinical myocardial toxicity. Given the involvement of immune checkpoints in many aspects of cardiovascular homeostasis, the mechanisms are probably multiple and interrelated (20). Therefore, the change in myocardial mechanics during PD1/PDL1 immunotherapy requires continuous monitoring. For the early recognition of ICI-related cardiac events, a basic assessment of cardiac status, including ECG, echocardiography, and troponin, should be considered before starting ICI therapy (21).

### 4.2 ICIs and right ventricular parameters

Previous studies found that the cutoff value of RVGLS and RVFWLS was –17.3 and –19.5%, respectively (10, 11). In pathological situations, right ventricular remodeling can occur when ventricular dilatation is produced by pressure overload (22). Because the interventricular septum is shared by the left ventricle and right ventricle, the RVGLS is influenced by both left and right ventricular function. It is reported that RVFWLS was the prior method to assess the right ventricle function of patients with pulmonary arterial hypertension (23). A slight change in right systolic function in patients with tumors could be identified through TAPSE (24). TAPSE less than 16 mm represents right ventricular systolic dysfunction (9). The study showed that compared with the baseline, TAPSE was significantly decreased post-ICIs. Kaplan–Meier curve analysis presented patients with reduced TAPSE had a higher incidence of death. The result coincidentally proved the important role of TAPSE to evaluate right ventricular systolic function.

### 4.3 ICIs and left ventricular parameters

Left and right ventricular toxicity can occur simultaneously (25). LVGLS and RVGLS were important indicators to assess the function of both chambers and can provide an early indicator of prognosis in patients with cancer. Our research demonstrated that LVGLS and RVGLS after ICI immunotherapy were significantly reduced than the baseline and lower than their normal range. Our results also proved that LV and RV functions were injured simultaneously after the administration of ICIs. Among the 55 patients, 15 patients were diagnosed with subclinical cardiac toxicity, according to the definition of LVGLS, reduced by more than 15%. Adverse cardiovascular events occurred in nine patients. The Kaplan–Meier curve analysis demonstrated that the criteria of LVGLS reduced by more than 15% were more sensitive than cardiac toxicity events to predict subclinical cardiac toxicity. LVEF is a load-dependent index, and preload, afterload, and myocardial contractility can all affect the LVEF value. All of these effects may lead to errors that affect the accuracy and sensitivity of LVEF (4, 12, 26).

### 4.4 ICIs and laboratory findings

Our early findings found no obvious increase in cTnI or NT-proBNP during follow-up, except in two cases. Laboratory parameters, including cTnI or NT-proBNP, were not optimum guidance for cardiotoxicity. Lena et al. (27) reported in EcoR Registry that the number of patients with increased cTnI or NT-proBNP was higher in patients with chemotherapy-induced cardiomyopathy than in patients without cardiotoxicity.

## 5 Limitations

There were several limitations of our study. First, a small number of patients were enrolled, and a single-center study was included. Further investigations in a multicenter and larger pool pattern will be needed to confirm the potential role of 2D-STI and 3D echocardiography in predicting ICI-related subclinical myocardial toxicity. Second, the heterogeneity of the patients was inevitable not only due to the small group of patients but also the complexity of individuals. Third, the follow-up study time was not sufficiently long, and the prognostic value of the different echocardiography indexes in the PD1/PDL1-related cardiotoxicity has not been thoroughly elucidated to date.

## 6 Conclusion

The cardiotoxicity of PD1/PDL1 immunotherapy can manifest as impaired left and right ventricular systolic function. LVGLS, RVGLS, and TAPSE are more sensitive indices for the early detection of subclinical cardiotoxicity.

## Data availability statement

The raw data supporting the conclusions of this article will be made available by the authors, without undue reservation.

## Ethics statement

The studies involving human participants were reviewed and approved by Medical Ethical Review Boards of the Tianjin Medical University Second Hospital. The patients/participants provided their written informed consent to participate in this study.

## Author contributions

HF was responsible for the manuscript concept, design, and definition of intellectual content. AX and MY were responsible for data analysis, statistical analysis, and manuscript preparation. XZ, GM, TW, and GZ were responsible for the literature search, clinical studies, and data acquisition. HH was responsible for manuscript editing and review. All authors contributed to the article and approved the submitted version.
